# Preventing chronic fatigue in Czech young athletes: The features description of the “SmartTraining” mobile application

**DOI:** 10.3389/fphys.2022.919982

**Published:** 2022-09-20

**Authors:** Martina Bernaciková, Michal Kumstát, Iva Burešová, Kateřina Kapounková, Ivan Struhár, Martin Sebera, Ana Carolina Paludo

**Affiliations:** ^1^ Department of Kinesiology, Faculty of Sports Studies, Masaryk University, Brno, Czechia; ^2^ Department of Health Promotion, Faculty of Sports Studies, Masaryk University, Brno, Czechia; ^3^ Incubator of Kinanthropology Research, Faculty of Sports Studies, Masaryk University, Brno, Czechia

**Keywords:** youth athlete, sport, fatigue, overtraining, mobile application

## Abstract

This study describes a beta version of a mobile application (app) that focuses on preventing chronic fatigue in Czech youth athletes. The first version of the SmartTraining app was developed for athletes as a way to prevent chronic fatigue *via* alertness and education. For alertness, a multistage process was developed using a combination of parameters about training responses, such as tiredness, well-being, heart rate, energy balance and psychological, and health-related aspects. According to the combination of the multistage parameter outcomes, the algorithm classifies the risk of fatigue based on semaphore light: green corresponds to low, yellow to moderate and red to high risk. The education presented in the app consisted of written and “animated videos” material about the variables involved in training, such as training demands and athletes’ responses, regeneration, nutrition and communication between athletes, coaches, and parents. Subsequently, a beta version of the app was created and freely available to download for Android or iOS mobile. The app can be used in daily routines to reduce the risk of chronic fatigue from inadequate training dose response. Prevention can minimise the risk of injury or physical and emotional burnout in youth. Informing athletes on how to carefully handle the training factors can improve athletes’ awareness of their performance and health status. Collaboration between sports scientists and the commercial sector allows for the efficient development of an easy-to-use and low-cost tool for use in sports settings. Future steps should be performed to validate the app’s accuracy in its alertness and in the efficiency of the educational process.

## 1 Introduction

Youth engagement in a sports programme that focuses on specialisation and the aspiration to reach a professional level is characterised by an intense training routine intended to optimise the athlete’s performance (Malina, 2010; Kliethermes et al., 2021). An inappropriate training dose (e.g., high intensity/volume) and insufficient recovery may result in maladaptive outcomes, such as decreased performance, increased risk of overuse-type injury (Kliethermes et al., 2021), overtraining (Matos et al., 2011; Burešová et al., 2021) or early dropout because of physical and emotional burnout (DiFiori et al., 2014). The occurrence of maladaptive responses becomes particularly important during adolescence, when youth athletes are undergoing different rates of biological maturation and psychological development (McKay et al., 2019).

To achieve peak performance, the training process involves a sufficient stimulus (training load) that can result in acute fatigue; with adequate recovery, performance super-compensation occurs, as described in the stimulus-fatigue-recovery-adaptation model (McGuigan, 2017). If the recovery process and the acute training stimulus is inadequate, the athlete may follow a fatigue continuum process, which comes with an impairment of performance, moving from acute fatigue, functional over-reaching to nonfunctional over-reaching and overtraining (McGuigan, 2017) (see the Supplementary Figure 1). Chronic fatigue and overtraining syndrome have been demonstrated to be an empirical concept and complicated to diagnose (Budgett, 2000; Weakley et al., 2022) because of the combination of complex factors, such as decreased performance, inadequate physiological and psychological responses and external variables. Moreover, the “Relative energy deficiency in sport syndrome (RED-S)”, which was recently introduced by the International Olympic Committee, shares similar etiological pathways and symptoms that are principally caused by the accumulation of inadequate recovery period. RED-S, however, is explicitly caused by chronically poor energy availability (Stellingwerff et al., 2021).

The prevalence of youth athletes who have experienced nonfunctional over-reaching or overtraining at least once during their sporting life reached up to 29% (Matos et al., 2011), 35% (Raglin et al., 2000), and 37% (Kenttä et al., 2001). Also, some have described that it is not just in elite athletes that these syndromes occur, showing that approximately 20% of youth playing at regional levels experienced the conditions at some point (Matos et al., 2011). Providing youth athletes with awareness about the training demands using an easy-to-use and low-cost tool can be a good approach for helping prevent chronic fatigue status. Therefore, the present study aimed to describe the development of a beta version of a mobile application (SmartTraining app) that can prevent chronic fatigue in Czech youth athletes. Considering the complex and multifactorial nature of chronic fatigue, the app focused on a combination of outcomes from the parameters involved in training responses and recovery. Likewise, the application also includes educational material that can be used to improve athletes’ knowledge about the training demands and recovery importance. The *SmartTraining app* was developed as part of a research project with the commercial sector to promote youth athlete health and performance.

## 2 Development description

The creation of the mobile application was based on the collaboration of sports researchers and coaches of youth athletes from Masaryk University, along with the Agency of Technology of the Czech Republic–TACR (Technologické Agentury České Republiky–ZETA), together with a private company (Code Creator). The project was designed to inform users about the prevention of chronic fatigue in youth based on a combination of parameters related to training responses. To achieve the proposed requirements, parameters related to chronic fatigue that were easy to measure in the youth’s daily routine were chosen. As mentioned, chronic fatigue in athletes is a complex combination of factors involving inadequate recovery and psychological outcome responses and external variables; therefore, the parameters selected were perceptual measures related to well-being and recovery, physiological responses, energy availability, and psychological responses (McGuigan, 2017; Weakley et al., 2022). Afterward, the development process of the mobile application started, which was performed closely by the leading researcher (sports scientist and coach) and software developer.

### 2.1 Fatigue prevention

**FIGURE 1:**
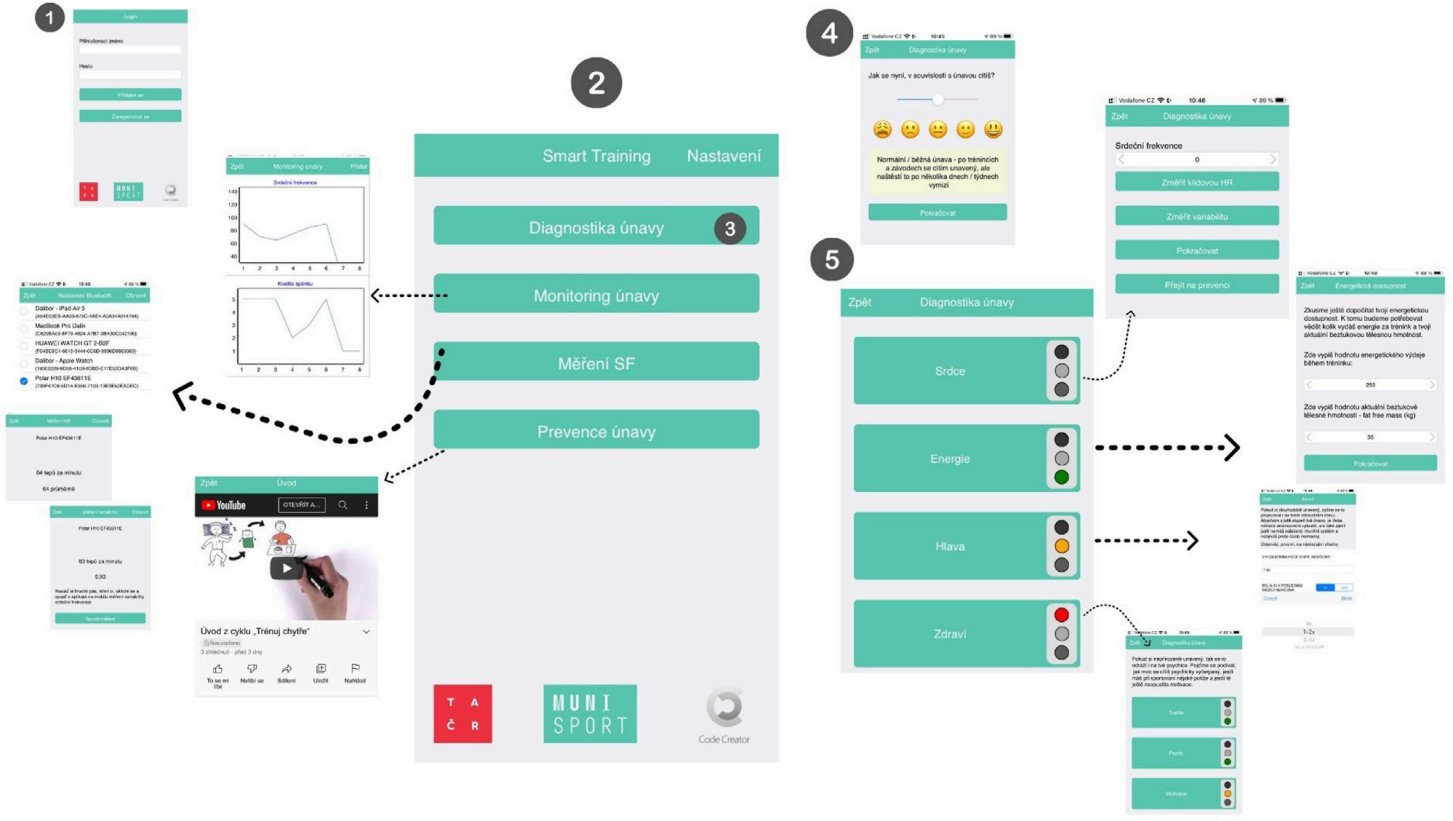
SmartTraining main window and stages of fatigue diagnosis. 1) access to the app; 2) main display; 3) fatigue diagnosis; 4) example of perceptual question and the options of answer; 5) fatigue parameters (heart, energy, head, and health) and the semaphore outcome.

The beta version of the SmartTraining app can be downloaded using either Android or iOS (instruction in the Supplementary Figure 2). The app was developed to be used by youth athletes; however, coaches can also have access to the educational part. The athlete’s profile allows inputting data, such as type of sport, sport training characteristics (weekly sessions and duration) and medical history. The main display presents the following activities: 1) fatigue diagnostics: multistage parameters related to training response; 2) fatigue monitoring: regular monitoring of parameters; 3) measuring heart rate: possibility to record the heart rate connected to an external device or input the values; and 4) fatigue prevention: access to written material (pdf) and animated videos with information about training demands and recovery (Figure 1).

The app activities are as follows.

#### 2.1.1 Fatigue diagnostics

The option *fatigue diagnostic* corresponds to fatigue prevention, which has been built as a multistage process involving the combination of the following training parameters: tiredness, well-being, heart rate, energy balance, psychological responses, and health-related measurements. This process corresponds to alertness based on the athletes’ answers for each parameter. The app has been built using an algorithm (based on categorical answers) to determine the risk of fatigue, here using a traffic light—green, orange or red—based on the continuum of fatigue (see Supplementary Figure 1). The first part corresponds to a perceptual question: How tired are you now? The athlete answers the question by selecting a Likert smiling face ranging from 1 (very bad) to 5 (very good), followed by a light traffic appearance. Answers of 3 or 4 points lead to a traffic light with orange colour, and the app recommends a prevention study (accessing the educational material). For answer 1, the light will show red, and the user will be guided to the second part by clicking the button “continue”. In the second part, the user continues to answer questions with a Likert smiling face [from 1 (very bad) to 5 (very good)] about sleep quality, muscle pain, and stress. If a single 1-point answer is selected, the traffic light continues to turn red, and the user will be led to the next level that discusses components such as the heart, energy, head, and health. If not, the light will turn orange, and athletes will be guided to access the educational videos.

**FIGURE 2:**
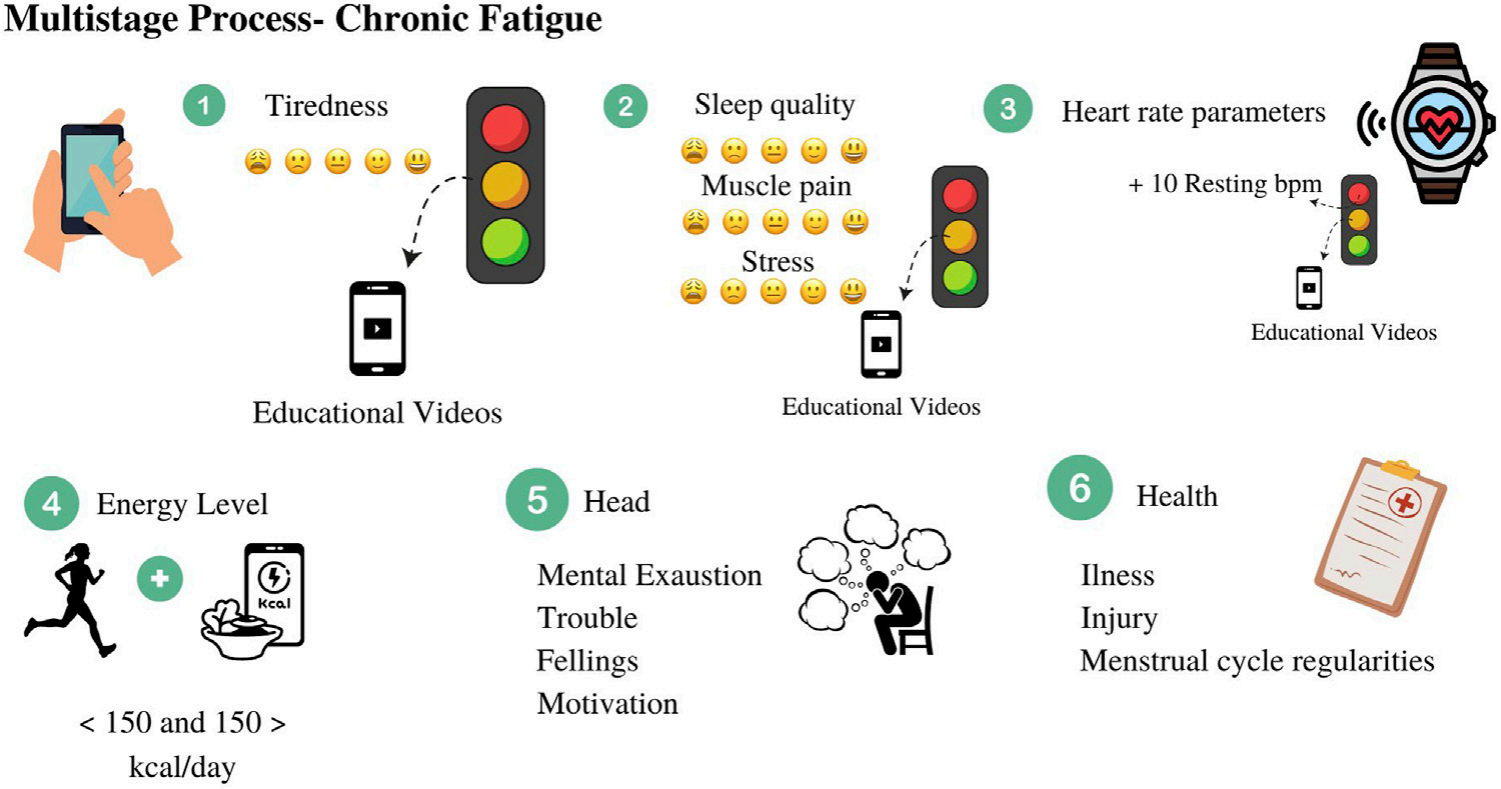
Training parameters assessed in a multisatge process of alertness for chronic fatigue.

The user will be oriented to gradually fill in all components during this process, which is described in Figure 2. The heart is related to the heart rate measurement (described in Section 2.1.3).

The energy level corresponds to the evaluation of the energy balance and energy availability of the athlete. Insufficient energy intake on days with high training demands (volume and/or intensity) can result in chronic fatigue if the condition is prolonged without appropriate recovery (Stellingwerff et al., 2021). The estimation of energy availability used in the app requires inputs of exercise energy expenditure for the training day, total daily energy intake and fat-free mass in kilograms. The total daily energy expenditure (TDEE) is determined by the calculation “Resting Energy × Physical Activity Level”. The estimation of resting energy expenditure (REE) is based on Watson et al.’s (2019) equation for the paediatric population [REE = 0.061 × Lean soft tissue (kg) − 0.138 × Sex (0 male, 1 female) + 2.41 (kcal/day)]. The physical activity level (PAL) has been coded as follows: 1) without training = PAL 1.6; 2) light training = PAL 1.8; and heavy training = PAL 2.0. The reference values for daily energy intake of relevant PAL are describes by the European Food Safety Authority (EFSA, 2017) for use in the general population. For energy intake, it is recommended that the user make a retrospective 24 h dietary record on the same training day previously selected. Also, it is recommended that the athlete perform the record data together with their parents. The app suggests electronic tools to calculate the daily energy intake (e.g., www.kaloricketabulky.cz; www.nutridata.cz).

If the final results (balance between predicted TDEE and recorded daily energy intake) differs more than 150 kcal/day from EFSA reference values, the user receives the following message: If the body receives more energy from food than it needs for a long time, it stores excess energy in fat reserves. Your weight increases disproportionately based on for your age and activity. You are at risk of being overweight. Results less than 150 kcal/day: If your body receives less energy than it needs through food for a long period of time, it disrupts healthy development and growth and fatigue increases. Your body will become more prone to injury. You may have a weakened immune system and become sick more often. Results between −149 and +149 kcal/day: Your energy intake corresponds to your energy output, that is, that your energy balance is fine. If you are ever not sure whether you are eating adequately for your training, feel free to come back to this measurement, or in the case of more demanding and frequent training, try the energy availability test in the next step. You can find more about proper and appropriate nutrition in the video or pdf document.

The cut-off point has been determined by the researcher (MK), here by considering previous literature and previous experience.

The head component focuses on the psychological side of the youth athlete. In this section, the athlete will answer how mentally exhausted they feel, if they have any problems with sports practice and if their keep motivation to continue to train and compete. The psychological component is separated into three domains: troubles, feelings, and motivation. The questions in which domain has been selected by the sports psychologist in the research team (IB) based on previous work (Květon et al., 2020; Burešová et al., 2021). The trouble domain asks, Have you had some problems during the last month in training or performance/competition? Twenty questions comprise this domain, with Yes or No options given. The feelings domain asks, How mentally exhausted do you feel? Twenty-three questions comprise this domain, with Yes or No options given. The motivation domain asks, Do you want to understand more about your motivation for sports? Twenty-two questions comprise this domain, with Yes or No options given.

The health component focuses on the athlete’s health status. Questions such as How often were you ill last year? Did you have injuries last year? are asked. For female athletes, questions about the menstrual cycle have been added.

After answering the multistage questions from the selected parameters, a light outcome based on semaphore color will appear. If the combined outcomes present a higher risk, a red light in the semaphore will be displayed together with a recommendation to the athlete to see a doctor and perform an appropriate analysis of biological components (e.g., hormonal, blood, urine measurement).

#### 2.1.2 Fatigue monitoring

The monitoring option allows the athletes to regularly record their responses to their training routines. Within monitoring, the athlete has the option to record parameters such as resting heart rate, sleep quality, muscle pain, stress, and fatigue sensation. For the heart rate measurement, it is possible to determine the average heart rate during training or resting using an external device (e.g., POLAR H10 chest strap). Chronic fatigue and overtraining have been related to a disturbance in resting heart rate outcomes (Djordjevic et al., 2013) and an impairment of perceptual responses of sleep quality, muscle pain, stress and fatigue domains, which comprise well-being parameters (McGuigan, 2017). If the combination of the heart rate and perceptual parameters is considered at risk of chronic fatigue long-term monitored, the athlete is recommended to answer the additional information about the parameters obtained in the multistage process.

#### 2.1.3 Heart measurement

This section corresponds to a heart rate measurement in a resting state. The user can either enter a current value of resting heart rate (e.g., measured using external devices such as a smartwatch or bracelet) or measure it using the app paired with a chest strap (e.g., POLAR H10 chest strap). In the “*settings*” menu, there is an option for *Bluetooth* to pair the app with a chest monitor. The app calculates only the average heart rate measured when resting. Normal values of athlete HR when resting, as measured on a day with no training and preferably in the morning, are required to register. During this process, if the athlete presents an HR resting higher than 10 bpm compared with their normal value, this is considered a risk; this has been established based on the researchers’ experience. Indeed, one of the first signs of overtraining is an increase in HR in resting (Bosquet et al., 2008); however, there is not a consensus on this matter (Fry et al., 1994). Because of this, the resting HR outcome is used together with the other training parameters.

#### 2.1.4 Fatigue prevention

**FIGURE 3:**
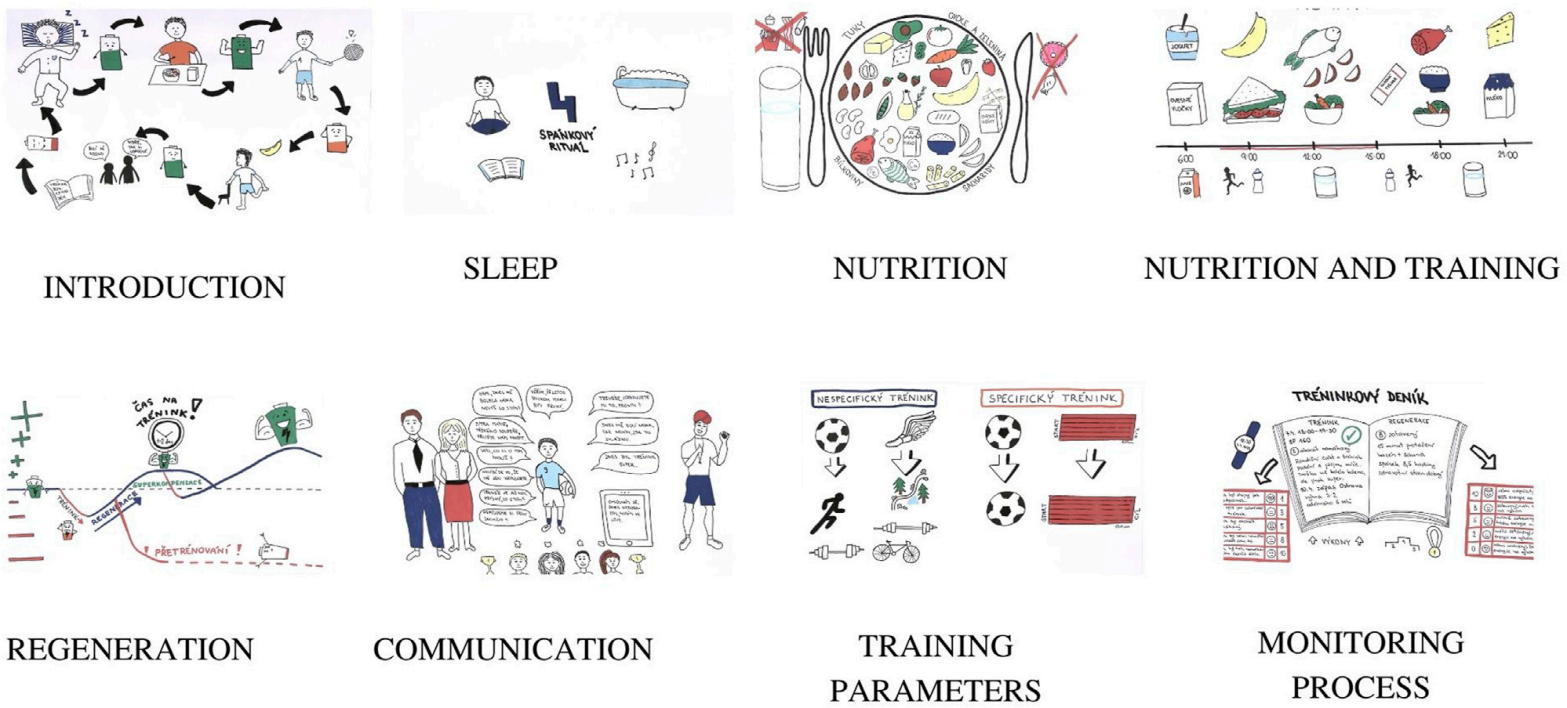
The educational part of the app: amination videos related to training demands and recovery (https://www.youtube.com/playlist?list=PL9yCtXX66neQPdsoMZl9gtCV7kz7jZuh0).

This part of the app is devoted to the educational process. The researcher team agreed with the importance of an education process for youth athletes regarding the parameters involved in sports training and chronic fatigue. Understanding the training demands and precautions that should be taken during the training routine could increase the chances of athletes adhering to preventive measures for chronic fatigue. These actions could help youth athletes have at minimal risk of fatigue, minimising the possibility of prolonging their fatigue status. The prevention component includes written materials (in pdf) and animated videos for the athletes with topics such as an introduction to training demands, sleep recommendations, nutrition, nutrition and training, regeneration, communication between the youth athletes, parents and coaches, principles of training and the importance of the monitoring process (Figure 3), as follows:

Introduction: An introductory video guiding the athlete through the daily training routine and the ways to prevent training overload.
Sleep: Descriptions of sleep quality as an important feature in youth athletes’ regeneration and sleep hygiene rules.
Nutrition: Introducing the basics of a healthy diet to which youth athletes should have adhered. In the video, a “plate” is displayed with the proportion of nutrients that a diet should consist of.
Nutrition and training: Demonstration of a day of intense training and competition, with recommendations about the time and quality of food intake.
Regeneration: The video points out the importance and reason for regeneration after training sessions. It provides an overview of basic regeneration methods that are possible to use, such as massages, saunas and cold baths.
Communication: The video shows how to improve communication between athletes and their coaches during the training process. Talking and listening are the keys to success in youth performance.
Training parameters: This video introduces the basic principles of sports training and the training process throughout the course of one’s sports career.
Monitoring: This describes the parameters that should be monitored during the training process. The video emphasises the importance of the training diary to record the training load and wellness status perceived by youth athletes.

For each topic, key information was developed by the researcher team based on academic evidence before then being adapted to a simple language to be better understood by the youth athletes. An easy itinerary is followed by an animation that is combined in a short video (up to 3:26 min). We have opted to present the content in animated videos to get attention and retention from users (Cookson et al., 2020). The prevention videos are displayed on the fourth option on the app’s main page. The athletes have access to pdf material and a YouTube page linked with the university’s channel that is exclusive to these users.

## 3 Discussion

The current article has described the process of developing a mobile application (SmartTraining) that focuses on preventing chronic fatigue in youth athletes. The app components have been chosen based on the academic and practical backgrounds of the research team, here considering prevention *via* alertness and education. To the best of our knowledge, no previous apps of a similar nature are currently available in the commercial market or described in the literature. The main idea was to develop an easy-to-use and low-cost tool that could minimise the risk of chronic fatigue and overtraining in youth athletes by improving their awareness about training demands. Educational programmes can play a key role in improving youth athletes’ knowledge about training parameters. An example of a similar form of education is the concussion programmes, which aim to inform youth athletes, parents and coaches about the concept of concussions (traumatic brain injury), the symptoms, prevention, and management (Williamson et al., 2014). The Centre for Diseases Control and Prevention (CDC) created an educational programme (Heads-up) based on training, videos, mobile apps and social media to educate those involved with youth athletes (Center for Disease Control and Prevention, 2022), which has presented a positive effect (Sarmiento et al., 2014). Similarly, the SmartTraining app aimed to integrate athletes and coaches into an educational experience about the possible causes of the occurrence of chronic fatigue in sports settings.

Chronicle fatigue and overtraining syndrome are severe conditions that can negatively affect athletes’ performance and well-being; however, there is little data in the literature that can provide evidence for the prospective nature of the variables that required for diagnosis (Budgett, 2000; Weakley et al., 2022). Recently, a review article has indicated that there are no studies meeting the criteria and definition of overtraining syndrome, showing that “the studies failed to provide evidence of changes in physical capacity from “healthy” to an overtrained state with chronic (more than 4 weeks) suppression of performance”. Indeed, follow-up time is important for diagnosis and can provide the best opportunities to have data from athletes; however, this process may be difficult because of the issues associated with data collection and valid tools used (Weakley et al., 2022). Certainly, when the training responses are monitored and integrated with an alertness system, as suggested in the app, methodological attention should be considered by looking at the measurement accuracy to detect the smallest changes (Schneider et al., 2018). Therefore, the SmartTraining app was developed to be used as the first step in preventing chronic fatigue in youth athletes. In the case of high risk, as identified by the parameters established in the app, the athlete should consult a doctor to perform an appropriate analysis of their biological components. Nonetheless, long-term monitoring of the chronic fatigue markers during an athlete’s sports life can be an ideal scenario to understand the dynamics of training dose response and for establishing possible prediction about it. In this case, mathematical modelling and artificial intelligence in the near future can help facilitate this process with more precision. Technological innovations should accelerate field-based measurements, helping monitor the athletes’ training process. The collaboration between sports scientists and commercial agencies can help in developing a more ecologically valid approach to personalised training prescriptions (Jonvik et al., 2022).

### 3.1 Limitations

The present study is the first part of a project focused on healthy youth athletes’ performance and has not yet been tested in the real world over a long-term period. Further steps should be taken to validate the applicability and usability of the app. In addition, the parameters chosen in the app for alertness consist of those that could be easy to self-report by the youth athletes in their daily routine. Thus, considering the complex factors involved in chronic fatigue, the app should be used together with a medical consultant in the case of a “the red alert”.

### 3.2 Future work

It is essential to perform a pedagogical usability evaluation of the SmartTraining app, here by considering multiple interfaces. Feedback from the users is necessary to improve the app, together with the research team, commercial agency, and designers’ experts. For the long-term use of the app, it is necessary to test the prototype to identify flaws and fix them. Furthermore, a validation of the prevention and alertness should be performed to understand the process that leads the youth athlete from “healthy” to “overtrained status”. Validation should involve a large dataset that considers various sports characteristics (individual vs. team sports) and athlete sex (female vs. male). Moreover, the potential negative consequences of actively monitoring certain parameters (e.g., counting calories) can trigger some harmful behaviour, such as eating disorders. As published elsewhere, some diet and fitness apps could trigger and exacerbate eating disorders symptoms by focusing heavily on quantification, promoting overuse (Eikey, 2021). To avoid this, the next version will minimise the risk by providing a more detailed nutritional programme, adding educational videos about the risk of eating disorders, as suggested in the ATHENA programme (Elliot et al., 2004).

The authors are open to collaboration with the scientific and commercial sectors in this matter.

## 4 Conclusion

The current project has aimed to develop a mobile application that can prevent chronic fatigue in youth athletes. The SmartTraining app is an initiative of mHealth app in the field of sports training in youth athletes, connecting science and practice by considering not only the development of an easy-to-use and low-cost tool, but also an educational approach for the youth athletes and their coaches. The app has the potential to be used in the practical field and in the youth athlete training routine, hence maximising the performance, fitness and health components and minimising the negative effect of intense training and inadequate recovery on the risk of injury or physical and emotional burnout. Future studies are suggested to validate the tool regarding the efficiency of the prevention process.

## Data Availability

All relevant data is contained within the article: The original contributions presented in the study are included in the article/Supplementary Materials, further inquiries can be directed to the corresponding authors.
