# Supplementary material for: Preventing chronic fatigue in Czech young athletes: The features description of the “SmartTraining” mobile application
Source: Front Physiol. 2022 Sep 20;13:919982. doi: 10.3389/fphys.2022.919982 (PMC9531124; doi:10.3389/fphys.2022.919982)
Supplement: Supplementary file 1 [file DataSheet1.docx]

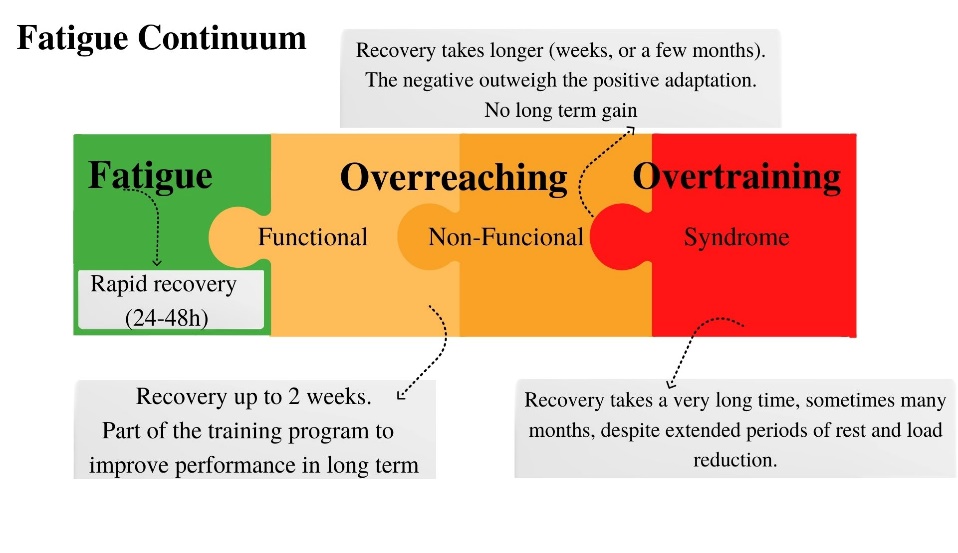


**Supplemental material. Figure 1.** Fatigue continuum and the classification risk of fatigue, updated from McGuigan, M. (2017).


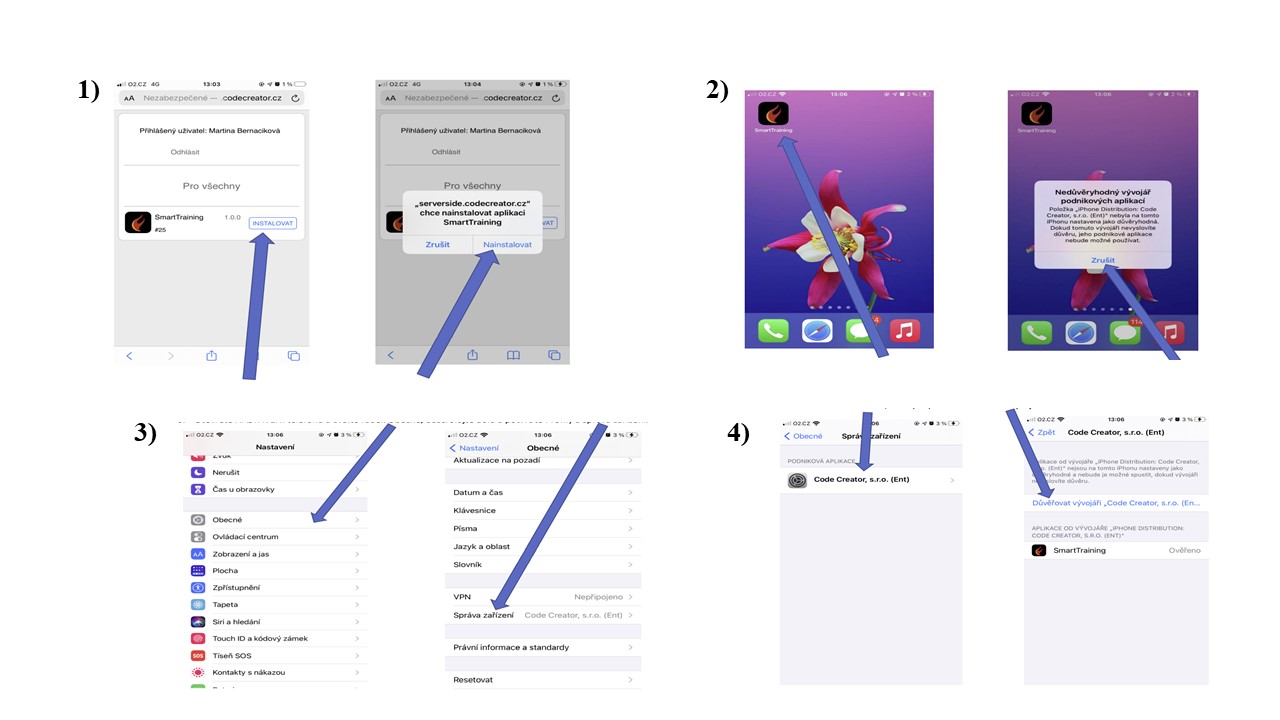


**Supplemental material. Figure 2.** Smart Training app download and installation process.

Follow these steps to install the iOS application:**1)** In Safari on your iOS device, open the page: <https://serverside.codecreator.cz/aplikace/apks/SmartTraining.apk>, click the INSTALL button, and confirm the prompt - select INSTALL. The application will be installed on the desktop of the iOS device. **2)**Click the SmartTraining application icon. A message appears stating that the application cannot be started. Confirm Cancel. **3)** Open Phone SETTINGS and select the General line, scroll down and confirm Profiles and device management. **4)** Click Code Creator, s.r.o. (Ent) and choose Trust Developer. Then start the application by clicking on the SmartTraining icon.
